# Predictors of Pathologic Complete Response and Its Prognostic Value in Early Breast Cancer: A Real-World Cohort Study

**DOI:** 10.3390/curroncol32110603

**Published:** 2025-10-28

**Authors:** Selami Bayram, Ali Murat Tatli, Muharrem Okan Cakir, Mustafa Ozdogan

**Affiliations:** 1 Department of Medical Oncology, Memorial Antalya Hospital, 07025 Antalya, Türkiye; alimurattat@hotmail.com (A.M.T.); ozdoganmd@yahoo.com (M.O.); 2 School of Life Science, Pharmacy and Chemistry, Kingston University London, London KT1 2EE, UK; m.okan@kingston.ac.uk

**Keywords:** breast neoplasms, early breast cancer, neoadjuvant therapy, pathologic complete response, prognosis, predictive biomarkers, HER2-positive breast cancer, triple-negative breast cancer, disease-free survival

## Abstract

**Simple Summary:**

We studied 200 people with early breast cancer who received treatments before surgery (chemotherapy and, when appropriate, anti-HER2 therapy). Our main goal was to learn which patients are most likely to have no invasive cancer found at surgery—a “pathologic complete response,” which is linked to better outcomes. Overall, 36% reached this outcome, most often in HER2-positive and triple-negative disease. Patients were more likely to clear the cancer if they had HER2-positive tumors, very low estrogen-receptor levels, a high tumor growth index, and a complete response on imaging before surgery. Those who cleared the cancer at surgery had a much lower chance of the cancer returning at five years (about 92% vs. 73% remained cancer-free). These results from routine practice can help doctors identify patients who may benefit from treatment escalation when cancer remains after surgery.

**Abstract:**

Background: Pathologic complete response (pCR) after neoadjuvant systemic therapy (NAST) is a key prognostic marker in early breast cancer (EBC), particularly in triple-negative (TNBC) and HER2-positive subtypes. However, real-world data on predictors of pCR and their impact on survival remain limited. Methods: We retrospectively analyzed 200 patients with stage II–III EBC treated with NAST at a single institution (2015–2023). Clinicopathologic variables and treatment characteristics were evaluated for association with pCR (ypT0/is ypN0), and histological regression was additionally assessed using the Miller–Payne scoring system. Multivariable logistic regression identified independent predictors. Disease-free survival (DFS) and overall survival (OS) were estimated using Kaplan–Meier methods. Results: Overall, 36.0% achieved pCR, with the highest rates in HER2-positive (65%) and TNBC (56%) subtypes. Independent predictors of pCR included HER2 positivity (OR 4.21, 95% CI 1.83–9.67, *p* < 0.001), high Ki-67 > 47.5% (OR 3.62, 95% CI 1.68–7.80, *p* = 0.001), ER < 10% (OR 2.77, 95% CI 1.18–6.50, *p* = 0.019), and radiologic complete response (OR 10.03, 95% CI 2.91–34.60, *p* < 0.001). At a median follow-up of 75 months, compared with non-pCR, patients achieving pCR had a significantly lower risk of recurrence (HR 0.16, 95% CI 0.04–0.70, *p* = 0.014) with 5-year DFS rates of 91.5% vs. 72.8%. For OS, pCR patients showed a lower risk of death (HR 0.33, 95% CI 0.07–1.49, *p* = 0.150), corresponding to 5-year OS of 92.2% vs. 87.0%, although this difference was not statistically significant. Conclusions: HER2 positivity, high Ki-67, low ER expression, and radiologic complete response are independent predictors of pCR. Achieving pCR strongly correlates with improved DFS but not OS, likely due to limited sample size and event number. These findings reinforce pCR as a surrogate endpoint in TNBC and HER2-positive disease and highlight the need for post-neoadjuvant escalation in non-pCR patients.

## 1. Introduction

Neoadjuvant systemic therapy (NAST) has become a cornerstone of care for biologically high-risk early breast cancer (EBC), enabling tumor downstaging, increasing breast-conserving surgery rates, and—critically—revealing in vivo chemosensitivity before definitive surgery [1,2]. In this setting, pathologic complete response (pCR) has been adopted as a key intermediate endpoint because patients who attain pCR generally experience substantially lower risks of recurrence and death compared with those with residual disease, especially in triple-negative (TNBC) and HER2-positive subtypes [3]. Contemporary international guidelines therefore recommend NAST for most stage II–III TNBC and HER2-positive tumors and selected higher-risk HR-positive/HER2-negative disease, both to maximize curative intent and to inform post-neoadjuvant therapy choices [1,2].

Across pooled trial datasets and meta-analyses, achieving pCR is strongly associated with improved event-free and overall survival in TNBC and HER2-positive breast cancer; the association is weaker and less frequent for HR-positive/HER2-negative tumors, in which endocrine therapy dominates long-term risk reduction [3,4].

Crucially, the clinical importance of neoadjuvant response has intensified with effective post-neoadjuvant escalation strategies for patients with residual invasive disease: capecitabine improved disease-free and overall survival in HER2-negative disease (particularly TNBC) in CREATE-X and adjuvant trastuzumab emtansine (T-DM1) halved the risk of invasive recurrence versus trastuzumab in HER2-positive residual disease in KATHERINE [5,6]. In parallel, adding pembrolizumab to platinum-taxane/anthracycline-based NAST followed by adjuvant pembrolizumab significantly increased pCR and event-free survival and, with mature follow-up, improved overall survival in high-risk stage II–III TNBC (KEYNOTE-522) [7]. Together, these data reinforce pCR as a clinically meaningful waypoint that stratifies subsequent therapy and prognosis.

A wide array of clinicopathologic, host, and treatment-related factors have been associated with pCR. Tumor biology (ER/PR/HER2 status, histologic grade, proliferation) is foundational; higher grade and elevated Ki-67 frequently track with higher pCR rates, particularly in non-luminal disease, although cut-offs and assay variability limit routine decision thresholds [2,8,9,10]. The tumor-immune microenvironment also matters: stromal tumor-infiltrating lymphocytes (TILs), standardized by the International TILs Working Group, consistently predict higher pCR and favorable outcomes—most notably in TNBC and increasingly in HER2-positive disease [11,12,13]. Germline BRCA1/2 status may further influence chemosensitivity, with several contemporary cohorts and meta-analyses reporting higher pCR rates—especially among BRCA1 carriers—though interactions with platinum use remain nuanced [14].

Therapy composition itself is a determinant of pCR. In TNBC, multiple randomized trials demonstrated that adding carboplatin to taxane-anthracycline backbones increases pCR rates (CALGB-40603; GeparSixto), with longer-term follow-up from BrighTNess trial supporting an event-free survival benefit attributable to carboplatin [8,9,15,16]. In HER2-positive disease, dual HER2 blockade with pertuzumab and trastuzumab plus chemotherapy robustly increases pCR (e.g., NeoSphere; TRYPHAENA), informing current standards and de-escalation research in pCR achievers [17,18]. Finally, the integration of immune checkpoint blockade in TNBC (KEYNOTE-522) further elevates pCR and survival, reshaping modern neoadjuvant algorithms and emphasizing the need to understand which patients derive the greatest benefit [7].

Despite these advances, real-world data identifying independent predictors of pCR across heterogeneous practice patterns and regimens remain limited, particularly from single-center cohorts outside large trial networks. Moreover, pCR definitions vary (e.g., ypT0 ypN0 vs. ypT0/is ypN0), and residual disease is heterogeneous, prompting complementary metrics such as the Residual Cancer Burden (RCB) index to refine prognostication [3,19]. Although RCB is widely validated, in our study histological regression was assessed using the Miller–Payne score due to its routine availability in our pathology workflow.

Against this backdrop, we conducted a retrospective observational study of patients with EBC treated with NAST at our institution to (i) estimate overall and subtype-specific pCR rates and (ii) identify clinicopathologic and treatment-related factors independently associated with pCR.

We hypothesized a priori that tumor subtype, higher grade/proliferation, and selected treatment components (platinum in TNBC, dual anti-HER2 therapy) would be associated with higher pCR rates, whereas higher baseline clinical stage would predict lower pCR. Biomarkers such as TILs and germline BRCA status, although not available in our cohort, have been reported as potential predictors in recent studies. We prespecified the ypT0/is ypN0 definition of pCR, consistent with CTNeoBC [3].

## 2. Materials and Methods

### 2.1. Study Design and Oversight

We conducted a single-center, retrospective observational cohort study at Memorial Antalya Hospital (Antalya, Türkiye). Consecutive patients with biopsy-proven, non-metastatic invasive breast cancer who received neoadjuvant systemic therapy (NAST) with curative intent and subsequently underwent definitive surgery between 2015 and the database-lock for this analysis were eligible. The institutional ethics committee approved the study; given the retrospective design and use of de-identified data, informed consent was waived in accordance with local policy. Reporting follows the STROBE guideline; a completed checklist is provided in the Supplement.

### 2.2. Participants: Eligibility Criteria

Inclusion criteria were female sex; histopathological confirmation of invasive breast carcinoma; clinical stage I–III disease at presentation; receipt of NAST (chemotherapy ± anti-HER2 therapy); and availability of complete pre-treatment, on-treatment, and post-surgical pathology/clinical records. Exclusion criteria were de novo metastatic disease at diagnosis and materially incomplete clinicopathologic information after data abstraction. Consecutive case identification from the institutional electronic health record minimized selection bias.

### 2.3. Imaging, Treatment, Surgery, and Pathology

Baseline and restaging imaging followed institutional routine and included PET-CT, breast/axillary ultrasound, mammography, and breast MRI as clinically indicated. Radiologic response categories (complete response [CR], partial response [PR], stable disease [SD], progressive disease [PD]) were abstracted from radiology reports (RECIST 1.1 where documented). NAST regimens were anthracycline- and/or taxane-based; platinum use and anti-HER2 therapy (trastuzumab with or without pertuzumab) were recorded when applicable. Anthracycline→taxane regimens were delivered either dose-dense every 2 weeks with mandatory G-CSF support or every 3 weeks at physician discretion. Definitive surgery consisted of breast-conserving surgery or mastectomy with sentinel lymph node biopsy and/or axillary dissection according to guideline-concordant indications.

Before NAST, axillary staging included ultrasound. Suspicious nodes underwent ultrasound-guided fine-needle aspiration (FNA) or core-needle biopsy when feasible; cytologic/histologic positivity defined cN+. Positive nodes were clipped whenever possible, and surgery included targeted axillary dissection (TAD) ± sentinel lymph-node biopsy according to institutional practice. Pathologic nodal response was reported as ypN.

Pathology was performed per institutional standard operating procedures. Estrogen receptor (ER) and progesterone receptor (PR) expression were quantified by immunohistochemistry and analyzed categorically using pre-specified thresholds aligned with your statistical plan (ER < 10% vs. ≥10%; PR < 20% vs. ≥20%). HER2 status was assessed by immunohistochemistry with reflex in situ hybridization for 2+ cases; categories included negative/1+, 2+ FISH−, 2+ FISH+, and 3+. Ki-67 was recorded as a continuous percentage; for analyses it was evaluated both at ≥20% vs. <20% and using a data-driven cut-point identified by ROC/Youden (see below). Stromal tumor-infiltrating lymphocytes (TILs) were not assessed and were therefore not analyzed. Histologic grade (Nottingham), lymphovascular invasion (LVI), and perineural invasion (PNI) were abstracted from final reports. In addition to pCR, tumor regression was also graded using the Miller–Payne (MP) system (MP1–MP5). This histological regression score provides a semi-quantitative measure of chemotherapy response by comparing cellularity in pre-treatment biopsy and post-treatment surgical specimens and has been correlated with survival outcomes in early breast cancer.

### 2.4. Outcomes

The primary endpoint was pathologic complete response (pCR), defined a priori as ypT0/is ypN0 at definitive surgery. Secondary endpoints included radiologic response category, breast-conserving surgery rate, disease-free survival (DFS; time from surgery to first locoregional or distant recurrence or death from any cause), and overall survival (OS; time from surgery to death from any cause), each censored at last known follow-up.

### 2.5. Variables and Data Sources

Abstracted variables included age, menopausal status, body mass index, clinical T/N stage, tumor laterality/quadrant, histologic type and grade, ER/PR/HER2, Ki-67, LVI, PNI, molecular subtype categorization, NAST regimen components (anthracycline, taxane, platinum), use of anti-HER2 therapy (trastuzumab/pertuzumab), number of chemotherapy cycles, and imaging response category. Data were obtained from the electronic health record, pathology information system, and institutional treatment records. Only cases with complete data for the primary endpoint and key covariates were included (complete-case analysis).

### 2.6. Statistical Analysis

Categorical variables were summarized as frequencies and percentages, and continuous variables as mean (SD), median (IQR), and range. Normality was assessed using the Shapiro–Wilk test. Between-group comparisons were performed using Pearson’s chi-square or Fisher’s exact test for categorical variables, and the Mann–Whitney U test for non-normally distributed continuous variables.

An optimal Ki-67 cut-off was determined using receiver operating characteristic (ROC) analysis with the Youden index. Receiver operating characteristic (ROC) curves were constructed to evaluate the discriminatory capacity of Ki-67 for pCR. The area under the curve (AUC) with 95% confidence intervals (CIs) was estimated using DeLong’s method and tested against the null AUC of 0.50. The optimal Ki-67 cut-off was chosen by maximizing Youden’s J; sensitivity and specificity at this threshold were reported. The selected threshold (47.5%) was then used to dichotomize Ki-67 in regression analyses. Predictors of pCR were first assessed in univariable analyses, and variables with *p* < 0.05 and/or clinical relevance were included in a multivariable binary logistic regression model. Odds ratios (ORs) with 95% confidence intervals (CIs) were reported.

Time-to-event endpoints (DFS, OS) were estimated using Kaplan–Meier methods and compared with the log-rank test. One-, three-, and five-year survival estimates were reported with 95% CIs. Two-sided *p* < 0.05 was considered statistically significant. Analyses were performed using Jamovi v2.6.44.

### 2.7. Data Protection

All data were de-identified prior to analysis and handled in accordance with institutional data-protection standards and the ethics approval.

## 3. Results

### 3.1. Patient Characteristics

A total of 200 patients with stage II–III breast cancer were included. The median age was 52 years (range, 27–84), with 51% premenopausal and 49% postmenopausal. The majority presented with stage IIB (52.5%) or IIIA (23.0%). Most tumors were grade 2 (74.0%), with a median Ki-67 index of 30% (IQR 10–40). ER and PR positivity were observed in 72.5% and 59.0% of patients, respectively, while HER2 positivity (IHC 3+ or 2+ FISH+) was present in 34.5%. Molecular subtype distribution was Luminal A (18.0%), Luminal B/HER2– (35.5%), Luminal B/HER2+ (21.0%), HER2-enriched (12.0%), and triple-negative (13.5%) (Table 1).

### 3.2. Treatment and Radiologic Response

Nearly all patients received anthracycline- (97.5%) and taxane-based (98.0%) regimens, while 13.5% received platinum. Anti-HER2 therapy was administered in 34% (13% trastuzumab, 21% dual blockade). Radiologic partial response was most frequent (80.0%), whereas complete response occurred in 12.0% and stable disease in 7.5%; progressive disease was rare (0.5%) (Table 1).

### 3.3. Adjuvant Therapy

Among patients who underwent surgery, adjuvant systemic therapies were administered according to molecular subtype and physician discretion. Endocrine therapy was delivered to 148 patients (74.0%), whereas 52 patients (26.0%) did not receive hormonal therapy. Thirteen patients (6.5%) received adjuvant capecitabine. Anti-HER2 adjuvant therapy was given to 65 patients (32.5%) with trastuzumab, and 6 patients (3.0%) received T-DM1 in the post-neoadjuvant setting. The majority (97.0%) did not receive T-DM1. These data reflect contemporary real-world practice during the study period (2015–2023).

### 3.4. Pathologic Response

Pathologic complete response (pCR; ypT0/is ypN0) was achieved in 72 patients (36.0%). According to the Miller–Payne regression score, 36.0% of patients achieved MP5 (complete loss of invasive tumor cells), whereas 11.5% remained MP1 (no significant reduction in cellularity). The remaining patients were distributed as MP2 (13.0%), MP3 (24.0%), and MP4 (15.5%). This distribution further illustrates the heterogeneity of treatment response beyond the binary definition of pCR.

**Figure 1:**
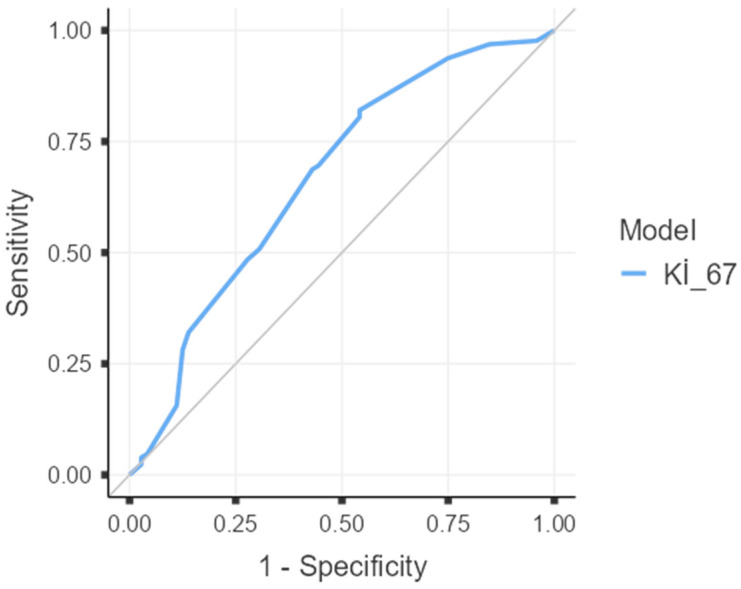
Receiver operating characteristic (ROC) curve of Ki-67 for prediction of pathologic complete response (pCR). AUC = 0.667 (95% CI 0.587–0.748; *p* < 0.001 by DeLong test). The optimal cut-off by Youden’s index was 47.5%, with sensitivity 0.82 and specificity 0.46. The diagonal line indicates no discrimination (AUC = 0.5).

On ROC analysis, Ki-67 showed modest discrimination for pCR: AUC 0.667 (95% CI 0.587–0.748; DeLong’s z = 4.07, *p* < 0.001). The optimal cut-off was 47.5%, yielding sensitivity 0.82 and specificity 0.46 for predicting pCR (Figure 1). This data-driven threshold was subsequently used in the multivariable models.

When comparing patients with and without pCR, ER and PR negativity, HER2 positivity, higher Ki-67 index, and radiologic complete response were significantly associated with pCR (all *p* < 0.01). Age, menopausal status, clinical T/N stage, histologic grade, LVI, and platinum use were not significantly associated (Table 2).

### 3.5. Survival Outcomes

**Figure 2:**
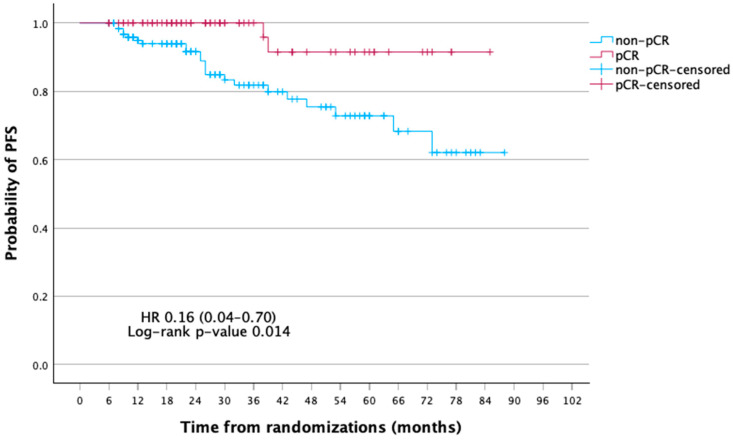
Kaplan–Meier curves of disease-free survival (DFS) according to pathologic complete response (pCR) status.

**Figure 3:**
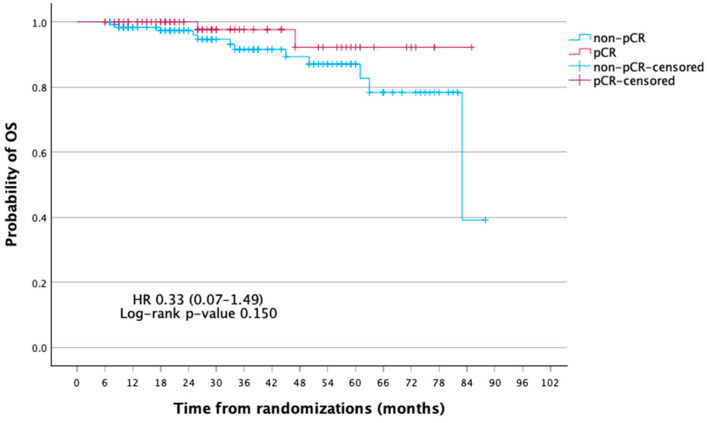
Kaplan–Meier curves of overall survival (OS) according to pathologic complete response (pCR) status.

At a median follow-up of 75 months, the mean disease-free survival (DFS) was significantly longer in patients achieving pCR compared with those without (81.0 vs. 70.0 months; 5-year DFS, 91.5% vs. 72.8%; *p* = 0.005). In Cox regression, pCR was associated with a markedly reduced risk of recurrence (HR 0.16, 95% CI 0.04–0.70, *p* = 0.014; log-rank *p* = 0.005). Five-year DFS was 91.5% for pCR vs. 72.8% for non-pCR. For OS, pCR patients had a lower risk of death (HR 0.33, 95% CI 0.07–1.49, *p* = 0.150; log-rank *p* = 0.131), with 5-year OS of 92.2% vs. 87.0%, though not significant (Table 3, Table 4 and Table 5; Figure 2 and Figure 3).

### 3.6. Multivariable Analysis

In multivariable logistic regression, independent predictors of pCR included HER2 positivity (OR 4.21, 95% CI 1.83–9.67, *p* < 0.001), high Ki-67 (>47.5%) (OR 3.62, 95% CI 1.68–7.80, *p* = 0.001), ER expression <10% (OR 2.77, 95% CI 1.18–6.50, *p* = 0.019), and radiologic complete response (OR 10.03, 95% CI 2.91–34.60, *p* < 0.001). Low PR (<20%) showed a nonsignificant trend (OR 1.96, 95% CI 0.83–4.61, *p* = 0.122). The model demonstrated good discrimination (AUC = 0.827) and overall classification accuracy of 75% (Table 6).

## 4. Discussion

In this single-center, real-world cohort, the overall pCR rate following neoadjuvant systemic therapy (NAST) was 36%, with the highest rates observed in HER2-positive and triple-negative breast cancer (TNBC) subtypes. Multivariable analysis confirmed HER2 positivity, high Ki-67 (>47.5%), low ER expression (<10%), and radiologic complete response as independent predictors of pCR. Consistent with prior studies, achieving pCR was associated with a markedly reduced risk of recurrence (HR 0.16, 95% CI 0.04–0.70). Although OS also favored pCR (HR 0.33, 95% CI 0.07–1.49), this difference did not reach statistical significance, most likely reflecting limited statistical power due to the small number of OS events despite long follow-up. These findings align with contemporary evidence underscoring pCR as a prognostic milestone, particularly in HER2-positive and TNBC cohorts [1,2,3,4].

Early breast cancer: ESMO Clinical Practice Guideline for diagnosis, treatment and follow-up. In HER2-positive disease, our ~65% pCR rate mirrors the results of NeoSphere and TRYPHAENA, where dual HER2 blockade markedly improved pCR [17,18]. In our study, dual trastuzumab–pertuzumab therapy was more frequently used among patients who achieved pCR, supporting the sustained relevance of dual blockade in real-world settings. Long-term results from KATHERINE further highlight the clinical implications of residual disease, showing that adjuvant T-DM1 halved recurrence risk in patients without pCR [6].

The prognostic significance of pCR observed in our cohort is consistent with pooled analyses such as CTNeoBC and subsequent meta-analyses, which confirmed strong associations with survival in TNBC and HER2-positive disease but weaker correlations in HR-positive/HER2-negative tumors [3,4]. Accordingly, current guidelines emphasize escalation strategies for patients with residual invasive disease: capecitabine in TNBC (CREATE-X), T-DM1 in HER2-positive breast cancer (KATHERINE), and perioperative pembrolizumab in TNBC (KEYNOTE-522) [5,6,7].

The predictive role of Ki-67 in our study is noteworthy. High Ki-67 independently predicted pCR, aligning with prior reports [2,8,9]. Our threshold choices should be interpreted within guideline frameworks. ASCO/CAP defines ER positivity at ≥1% with an ‘ER-low’ category (1–10%), while international Ki-67 working groups and expert panels (e.g., St. Gallen) emphasize inter-laboratory variability and consider only low (≈≤5%) and high (≈≥30%) ranges as robust for clinical decision-making. In that context, our ROC-derived Ki-67 cut-point (47.5%) is best viewed as real-world and hypothesis-generating rather than universal [20,21,22]. Consistent with prior studies, Ki-67 alone demonstrated only modest discriminatory capacity (AUC 0.667), whereas our multivariable model achieved good discrimination (AUC 0.827), supporting the use of composite predictors rather than single biomarkers. Similarly, low ER expression (<10%) was strongly associated with pCR, consistent with the biological aggressiveness and chemotherapy sensitivity of ER-low tumors. Stromal tumor-infiltrating lymphocytes (TILs), although not assessed in our cohort, have emerged as validated predictive and prognostic biomarkers, particularly in TNBC and increasingly in HER2-positive disease [11,12,13]. In addition, germline BRCA1/2 status was not available, despite its recognized role as a predictive and prognostic factor in recent studies [14]. The absence of these biomarkers represents a limitation and underscores the need for future cohorts incorporating standardized immune and genetic profiling to refine response prediction and survival stratification.

Radiologic complete response was the strongest independent predictor of pCR in our analysis (OR~10). This finding is supported by systematic reviews and meta-analyses demonstrating that breast MRI has high specificity for predicting pCR, especially in TNBC and HER2-positive subtypes [3,19]. Integration of imaging with pathologic endpoints may refine surgical decision-making and axillary management in the neoadjuvant setting.

Inflammatory indices such as neutrophil-to-lymphocyte ratio (NLR) and pan-immune-inflammation value (PIV) were not associated with pCR in our series. This is consistent with heterogeneous and inconclusive evidence in the literature, where these systemic markers show prognostic but not consistently predictive value for pCR [10,23,24]. Their utility may depend on subtype-specific analyses and dynamic changes during therapy.

As expected, HR-positive/HER2-negative tumors in our cohort had low pCR rates (~8–15%), in line with previous data [3,4]. In this subtype, long-term outcomes are primarily driven by endocrine therapy and, more recently, by adjuvant CDK4/6 inhibitors such as abemaciclib (monarchE) and ribociclib (NATALEE), which demonstrated significant improvements in invasive DFS [1,2]. Thus, pCR is of limited utility as a surrogate endpoint in this group, reinforcing the importance of biomarker-guided escalation strategies beyond neoadjuvant response. Residual Cancer Burden (RCB) scoring was not available in our pathology workflow during the study period; therefore, we used the Miller–Payne regression system (MP1–MP5), which, although less comprehensive as it does not include nodal involvement, provides a validated histological measure of chemotherapy response and has been correlated with survival outcomes in early breast cancer [25].

Adjuvant systemic therapies may have also influenced long-term outcomes in our cohort. Specifically, endocrine therapy was widely used in HR-positive disease, while a minority of TNBC patients received capecitabine and a subset of HER2-positive patients received trastuzumab or T-DM1. Although our analysis focused on predictors of pCR, differences in adjuvant treatments likely contributed to survival outcomes and may partially confound the observed association between pCR and DFS/OS.

Although pCR associated with superior DFS in our cohort, the difference in OS did not reach statistical significance (*p* = 0.150). This likely reflects limited event numbers and immature OS information in some subgroups, raising the possibility of type-II error. Longer follow-up and/or multi-center validation will better define OS effects.

Contribution of this work; In a long-followed single-center real-world cohort, we (i) quantified subtype-specific pCR under heterogeneous routine regimens; (ii) identified independent predictors—including radiologic complete response, HER2 positivity, lower ER, and a ROC-derived high Ki-67—with strong model discrimination (AUC 0.827); and (iii) linked pCR to significantly improved DFS while transparently acknowledging limited OS power. These findings support pragmatic risk stratification and can inform post-neoadjuvant decision pathways in routine practice.

### 4.1. Limitations

This study has several limitations. First, it was a retrospective, single-center analysis, which carries inherent risks of selection and information bias. Second, the study period (2015–2023) preceded the widespread adoption of immune checkpoint inhibitors in TNBC and standardized use of platinum in this setting, as well as the integration of T-DM1 into the adjuvant management of residual HER2-positive disease. Thus, our findings reflect historical real-world practice and may not fully represent outcomes in the contemporary treatment era. Third, key biological variables such as tumor-infiltrating lymphocytes (TIL), germline BRCA status, and Residual Cancer Burden (RCB) were not uniformly available retrospectively; we therefore used Miller–Payne for pathologic response where applicable. Given the known associations of TIL/BRCA/RCB with pCR and outcomes, we plan standardized prospective capture in future cohorts. Fourth, the proportion of patients receiving platinum was relatively low, and no patients received neoadjuvant immunotherapy, which may have affected observed pCR rates. Finally, although median follow-up exceeded 6 years—adequate for capturing clinically meaningful disease-free and overall survival events—statistical power to detect overall survival differences remained limited by sample size.

### 4.2. Clinical Implications and Future Directions

Despite these limitations, the present study provides clinically relevant insights. HER2 positivity, high Ki-67, low ER expression, and radiologic complete response emerged as independent predictors of pCR. These findings may help refine patient counseling, guide clinical decision-making, and support stratification in future clinical trials. The strong association between pCR and disease-free survival reinforces the role of pCR as a meaningful early efficacy endpoint, particularly in TNBC and HER2-positive subtypes.

From a therapeutic perspective, patients who fail to achieve pCR remain candidates for evidence-based post-neoadjuvant escalation strategies. These include adjuvant capecitabine in TNBC (CREATE-X), T-DM1 in HER2-positive disease with residual tumor (KATHERINE), and perioperative pembrolizumab in high-risk TNBC (KEYNOTE-522). Integration of such strategies into routine practice is critical to improve long-term outcomes in patients with residual disease.

Our data also highlight the need for better incorporation of predictive biomarkers—including stromal TILs, RCB scoring, and germline BRCA testing—into daily practice, as these have been shown to provide additional prognostic and predictive information [11,12,13,14,19]. Future research should prioritize prospective, multicenter real-world cohorts that incorporate contemporary regimens, including immunotherapy and targeted agents, as well as standardized biological markers. Our findings may help refine patient counseling and guide the design of future multicenter registries aimed at validating predictive models and prognostic tools in real-world settings.

## Figures and Tables

**Table 1:** Baseline demographic, clinical, and laboratory characteristics of patients.

		Median	Min–Max			Mean ± sd/n%		
Age		52	27	–	84	52.76	±	11.41
Menopausal status	Premenopausal					102		51.0%
	Postmenopausal					98		49.0%
Clinical Stage	2A					13		6.5%
	2B					105		52.5%
	3A					46		23.0%
	3B					22		11.0%
	3C					14		7.0%
cT stage	T1					21		10.5%
	T2					132		66.0%
	T3					23		11.5%
	T4					24		12.0%
cN stage	N0					4		2.0%
	N1					145		72.5%
	N2					37		18.5%
	N3					14		7.0%
Grade	Grade 1					6		3.0%
	Grade 2					148		74.0%
	Grade 3					46		23.0%
Ki-67		30	1	–	90	33.52	±	23.44
ER status	negative					55		27.5%
	positive					145		72.5%
ER category	<%10					61		30.5%
	≥%10					139		69.5%
PR status	negative					82		41.0%
	positive					118		59.0%
PR category	<%20					110		55.0%
	≥%20					90		45.0%
HER2 status	Neg					74		37.0%
	1+					18		9.0%
	2+ FISH−					39		19.5%
	2+ FISH+					14		7.0%
	3+					55		27.5%
Molecular subtype	Luminal-a					36		18.0%
	Luminal-b her2 neg					71		35.5%
	Luminal-b her2 pos					42		21.0%
	Her2 positive					24		12.0%
	Triple negative					27		13.5%
NLR		2.3	1	–	13	2.64	±	1.35
PIV		345	80	–	2276	430.5	±	296.3
CEA	≤5					185		92.5%
	>5					15		7.5%
CA15.3	≤30					183		91.5%
	>30					17		8.5%
Anthracycline-based chemo	no					5		2.5%
	yes					195		97.5%
Taxane-based chemo	no					4		2.0%
	yes					196		98.0%
Neoadjuvant Anti-HER-2 agent	no					132		66.0%
	trastuzumab					26		13.0%
	trastuzumab plus pertuzumab					42		21.0%
Neoadjuvant platin	no					173		86.5%
	yes					27		13.5%
Radiologic response	Progressive disease					1		0.5%
	Stable disease					15		7.5%
	Partial response					160		80.0%
	Complete response					24		12.0%
ypT	ypT0/is					78		39.0%
	ypT1					71		35.5%
	ypT2					42		21.0%
	ypT3					8		4.0%
	ypT4					1		0.5%
ypN	ypN0					115		57.5%
	ypN1					55		27.5%
	ypN2					21		10.5%
	ypN3					9		4.5%
Miller-Payne Regression Score	MP1					23		11.5%
	MP2					26		13.0%
	MP3					48		24.0%
	MP4					31		15.5%
	MP5					72		36.0%
pCR	no					128		64.0%
	yes					72		36.0%
Adjuvant ET	no					52		26.0%
	yes					148		74.0%
Adjuvant Capesitabine	no					187		93.5%
	yes					13		6.5%
Adjuvant Trastuzumab	no					135		67.5%
	yes					65		32.5%
Adjuvant TDM-1	no					194		97.0%
	yes					6		3.0%

**Table 2:** Comparison of clinical, laboratory, and pathological parameters between pCR and non-pCR groups.

		Non-pCR (n: 128)				pCR (n: 72)				*p*	
		Mean	±	sd/n%	Median	Mean	±	sd/n-%	Median		
Age		51.99	±	11.66	51.5	54.11	±	10.89	52.5	0.208	t
Menopausal status	Premenopausal	66		51.60%		36		50.00%		0.832	X^2^
	Postmenopausal	62		48.40%		36		50.00%			
cT stage	T1	13		10.20%		8		11.10%		0.359	X^2^
	T2	80		62.50%		52		72.20%			
	T3	18		14.10%		5		6.90%			
	T4	17		13.30%		7		9.70%			
cN stage	N0	3		2.30%		1		1.40%		0.395	X^2^
	N1	88		68.80%		57		79.20%			
	N2	28		21.90%		9		12.50%			
	N3	9		7.00%		5		6.90%			
Grade	Grade 1	6		4.70%		0		0.00%		0.074	
	Grade 2	97		75.80%		51		70.80%			
	Grade 3	25		19.50%		21		29.20%			
Ki-67		28.24	±	20.42	25	42.92	±	25.57	40	<0.001	m
ER status	negative	21		16.40%		34		47.20%		<0.001	X^2^
	positive	107		83.60%		38		52.80%			
ER category	<%10	23		18.00%		38		52.80%		<0.001	X^2^
	≥%10	105		82.00%		34		47.20%			
PR status	negative	38		29.70%		44		61.10%		<0.001	X^2^
	positive	90		70.30%		28		38.90%			
PR category	<%20	55		43.00%		55		76.40%		<0.001	X^2^
	≥%20	73		57.00%		17		23.60%			
HER2 status	0	60		46.90%		14		19.40%		<0.001	X^2^
	1+	13		10.20%		5		6.90%			
	2+ FISH-	31		24.20%		8		11.10%			
	2+ FISH+	8		6.30%		6		8.30%			
	3+	16		12.50%		39		54.20%			
Molecular subtype	Luminal-a	33		25.80%		3		4.20%		<0.001	X^2^
	Luminal-b her-2 neg	60		46.90%		11		15.30%			
	Luminal-b her-2 pos	15		11.70%		27		37.50%			
	Her-2 positive	8		6.30%		16		22.20%			
	Triple negative	12		9.40%		15		20.80%			
NLR		2.64	±	1.18	2.35	2.64	±	1.61	2.28	0.52	m
PIV		428.1	±	281.8	353	434.7	±	322.4	341	0.998	m
CEA	≤5	119		93.00%		66		91.70%		0.737	X^2^
	>5	9		7.00%		6		8.30%			
CA15.3	≤30	118		92.20%		65		90.30%		0.642	X^2^
	>30	10		7.80%		7		9.70%			
Anthracycline-based chemo	no	4		3.10%		1		1.40%		0.656	X^2^
	yes	124		96.90%		71		98.60%			
Taxane-based	no	3		2.30%		1		1.40%		0.999	X^2^
chemo	yes	125		97.70%		71		98.60%			
Neoadjuvant Anti-HER-2 agent	no	105		82%		27		37.50%		<0.001	X^2^
	trastuzumab	13		10.20%		13		18.10%			
	trastuzumab plus pertuzumab	10		7.80%		32		44.40%			
Neoadjuvant platin	no	111		86.70%		62		86.10%		0.904	X^2^
	yes	17		13.30%		10		13.90%			
Radiologic response	Progressive disease	1		0.80%		0		0%		<0.001	X^2^

t Independent Samples *t* test/m Mann–Whitney U test/X^2^ Chi-square test (Fischer test).

**Table 3:** Kaplan–Meier estimates of DFS and OS according to pCR status.

Outcome	Group	Mean Survival (Months, 95% CI)	1-Year Survival %	3-Year Survival %	5-Year Survival %	*p*-Value
DFS	Non-pCR	70.0 (63.6–76.5)	94.9	81.8	72.8	0.014
	pCR	81.0 (75.8–86.3)	100	100	91.5	
OS	Non-pCR	76.6 (70.9–82.2)	98.3	91.5	87.0	0.150
	pCR	81.5 (76.8–86.3)	100	97.6	92.2	

DFS = disease-free survival; OS = overall survival; pCR = pathologic complete response; CI = confidence interval. Data are presented as percentages unless otherwise specified. Mean survival times are given with 95% CIs. *p*-values refer to log-rank test comparisons between pCR and non-pCR groups. A two-sided *p* < 0.05 was considered statistically significant.

**Table 4:** Summary of PFS outcomes by pCR status (events, mean survival, HR, and log-rank *p*-value).

	Non-pCR	pCR
Events n/N (%)	22/128 (17.19)	2/72 (2.78)
Mean PFS, months (%95 CI)	70.02 (63.58–76.47)	81.04 (75.78–86.29)
HR (%95 CI)	HR 0.16 (0.04–0.70)	
Log-rank *p*-value	0.014	

**Table 5:** Summary of OS outcomes by pCR status (events, mean survival, HR, and log-rank *p*-value).

	Non-pCR	pCR
Events n/N (%)	12/128 (9.37)	2/72 (2.78)
mOS, months (%95 CI)	76.56 (70.96–82.15)	81.53 (76.81–86.26)
HR (%95 CI)	HR 0.33 (0.07–1.49)	
Log-rank *p*-value	0.150	

**Table 6:** Multivariable logistic regression analysis of predictors of pathologic complete response (pCR).

Predictor	β (SE)	z	*p*-Value	OR	95% CI
HER2 positive (vs. negative)	1.43 (0.42)	3.38	<0.001	4.21	1.83–9.67
Ki-67 > 47.5% (vs. ≤47.5%)	1.28 (0.39)	3.28	0.001	3.62	1.68–7.80
ER < 10% (vs. ≥10%)	1.01 (0.43)	2.34	0.019	2.77	1.18–6.50
PR < 20% (vs. ≥20%)	0.67 (0.43)	1.55	0.122	1.96	0.83–4.61
Radiographic complete response (yes vs. no)	2.30 (0.63)	3.65	<0.001	10.03	2.91–34.60

Notes: β = regression coefficient; SE = standard error; OR = odds ratio; CI = confidence interval; pCR = pathologic complete response. *p*-values are derived from Wald z tests. A two-sided *p* < 0.05 was considered statistically significant. Ki-67 was dichotomized at 47.5%, the optimal threshold identified by ROC analysis using Youden’s index (see Figure 1).

## Data Availability

The datasets generated and/or analyzed during the current study are not publicly available due to institutional data protection policies but are available from the corresponding author on reasonable request.
